# A Three-Dimensional Printed Foot Orthosis for Flexible Flatfoot: An Exploratory Biomechanical Study on Arch Support Reinforcement and Undercut

**DOI:** 10.3390/ma14185297

**Published:** 2021-09-14

**Authors:** Ka-Wing Cheng, Yinghu Peng, Tony Lin-Wei Chen, Guoxin Zhang, James Chung-Wai Cheung, Wing-Kai Lam, Duo Wai-Chi Wong, Ming Zhang

**Affiliations:** 1 Department of Biomedical Engineering, Faculty of Engineering, The Hong Kong Polytechnic University, Hong Kong 999077, China; ka-wing-vicky.cheng@connect.polyu.hk (K.-W.C.); 18041923r@connect.polyu.hk (Y.P.); tony.l.chen@connect.polyu.hk (T.L.-W.C.); guo-xin.zhang@connect.polyu.hk (G.Z.); james.chungwai.cheung@polyu.edu.hk (J.C.-W.C.); 2 The Hong Kong Polytechnic University Shenzhen Research Institute, Shenzhen 518057, China; 3 Guangdong Provincial Engineering Technology Research Center for Sports Assistive Devices, Guangzhou Sport University, Guangzhou 510000, China; gilbertlam@li-ning.com.cn; 4 Department of Kinesiology, Shenyang Sport University, Shenyang 110102, China; 5 Li Ning Sports Science Research Center, Li Ning (China) Sports Goods Company, Beijing 101111, China

**Keywords:** pes planus, pes planovalgus, flexible flatfoot, pronation, customized insole, kinematics, plantar pressure, rapid prototyping

## Abstract

The advancement of 3D printing and scanning technology enables the digitalization and customization of foot orthosis with better accuracy. However, customized insoles require rectification to direct control and/or correct foot deformity, particularly flatfoot. In this exploratory study, we aimed at two design rectification features (arch stiffness and arch height) using three sets of customized 3D-printed arch support insoles (R+U+, R+U−, and R−U+). The arch support stiffness could be with or without reinforcement (R+/−) and the arch height may or may not have an additional elevation, undercutting (U+/−), which were compared to the control (no insole). Ten collegiate participants (four males and six females) with flexible flatfoot were recruited for gait analysis on foot kinematics, vertical ground reaction force, and plantar pressure parameters. A randomized crossover trial was conducted on the four conditions and analyzed using the Friedman test with pairwise Wilcoxon signed-rank test. Compared to the control, there were significant increases in peak ankle dorsiflexion and peak pressure at the medial midfoot region, accompanied by a significant reduction in peak pressure at the hindfoot region for the insole conditions. In addition, the insoles tended to control hindfoot eversion and forefoot abduction though the effects were not significant. An insole with stronger support features (R+U+) did not necessarily produce more favorable outcomes, probably due to over-cutting or impingement. The outcome of this study provides additional data to assist the design rectification process. Future studies should consider a larger sample size with stratified flatfoot features and covariating ankle flexibility while incorporating more design features, particularly medial insole postings.

## 1. Introduction

Flatfoot, also known as pes planus, is a foot deformity characterized by the flattening or collapse of the medial longitudinal arch and may manifest over-pronation, hindfoot eversion, forefoot abduction, and midfoot instability. Flexible flatfoot is the most common type, in which the arch could be reformed in non-weight-bearing conditions [1]; it affects 13.6% and 17.1% of adults and children [2,3]. Although flatfoot is usually asymptomatic, some patients may experience pain, swelling, intolerance, or gait dysfunction in severe cases [1,4,5]. Flatfoot is also linked to plantar fasciitis, knee osteoarthritis, and other foot deformities [6,7].

Conservative interventions could reduce the pain and prevent the progression of the deformity [8], whereas orthoses or footwear modifications are often prescribed. In cases of flatfoot with instability, ankle-foot orthosis (AFO) can control and stabilize the foot-ankle complex and reduce the load on the posterior tibial tendon and midfoot ligaments [9,10]. The University of California Berkeley Laboratories (UCBL) foot orthosis, made of a hard plastic shell with a heel cup and arch support, can maintain the medial longitudinal arch and keep the calcaneus in a neutral position [11]. In any case, depending on the patient’s condition after assessment, orthotists could also select appropriate materials to design customized orthosis with different components, such as arch support, metatarsal pad, heel wedge, and medial posting, etc., [11].

The effects of orthotic interventions have been evaluated by numerous biomechanical research, the majority of them focusing on the plantar pressure measurement and kinematic and kinetic parameters over walking gait [12,13]. For example, Tang, et al. [14] evaluated the use of forefoot medial posting insoles in flexible flatfoot patients and found that they could redistribute the plantar pressure and correct the rearfoot valgus. Su, et al. [15] tested different combinations of arch support height and insole material stiffness and found they can help attenuate peak pressure and control excessive pronation. In short, biomechanical evidence has demonstrated the effectiveness of traditional orthotic insoles and direct design optimization.

The advancement of additive manufacturing (or 3D printing) facilitates nascent applications and revolutionizes the orthotics industry and healthcare. The technology can digitize the traditional hand-crafted manufacturing process and designs with controlled precision [16,17]. Along with the advancement of 3D scanning and other imaging techniques, foot morphology could be acquired for customization and better fit in orthotic designs [16,18]. Moreover, 3D printing can vary the material characteristics by using different infill rates, printing textures, or patterns that change the mode of foot support for therapeutic purposes [18]. To this end, we proposed utilizing the strengths of 3D printing technology on foot shape customization and material characterization. The objective of this study was to develop 3D printed customized orthotic insoles with reinforced arch support via infill rate for flatfooted individuals. In this exploratory study, the biomechanical performance of this orthotic design was evaluated and compared to that without reinforcement; and to that with/without an undercut over the arch height, with the intention of evaluating the sensitivity of these key design features.

## 2. Materials and Methods

### 2.1. Participant Recruitment

We recruited ten collegiate participants with flexible flatfoot via convenience sampling in this pilot study. Their flatfooted condition was verified by the plantar arch index ≥ 0.9 [19,20]. Participants were excluded if they had a history of neuromuscular, vascular diseases, or other foot deformities or problems that were unassociated with flatfoot. They were also excluded if they had a lower limb surgery in the past six months. The participants also did not present or report any spinal deformities. The study protocol and ethical considerations were approved by the institutional review board (Reference No.: HSEARS20201209001). Every participant received a verbal and written explanation of the research and signed informed consent before the start of the experiment.

The ten participants comprised four males and six females with a mean age of 20.4 (SD: 0.9). Their height and bodyweight were 1.66 m (SD: 0.68) and 59.1 kg (SD: 6.0), respectively. Their foot size was 25.3 cm (SD: 1.6) with a plantar arch index of 1.17 (SD: 0.2) and navicular drop height of 1.2 cm (SD: 0.2).

### 2.2. Foot Shape Acquisition

**Figure 1:**
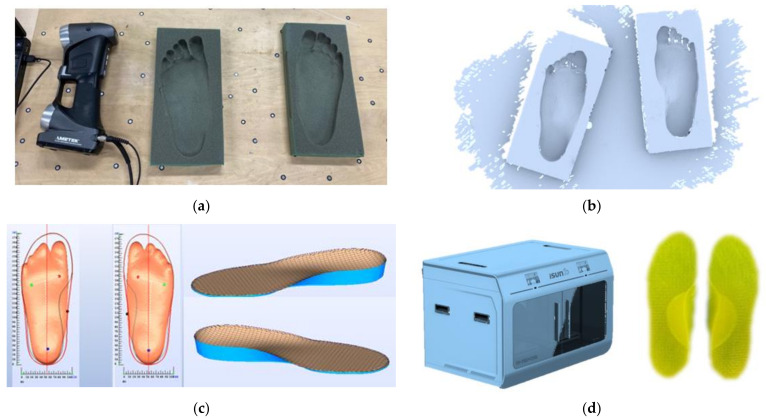
Workflow for fabricating the 3D customized orthotic insoles. (**a**) Foot shape acquisition through stepping onto the foam box. (**b**) Raw data of the digitized foot shape of the foam box by the 3D scanner. (**c**) Generating the orthotic design based on the foot shape using computer-aided design software, isoleCAD. (**d**) Fabrication of the 3D printed insole.

The 3D plantar contour data of the participants were needed for the customized fabrication. A foam impression box was used to take a cast of the participants’ feet according to the sitting protocol [21]. The feet of the participants were held in a subtalar joint neutral position as it was pushed into a standard foam impression box by the orthotist when the participants were sitting [21]. As shown in Figure 1a, the foam impression box was placed on a flat surface with reflective markers to calibrate the 3D scanner, HandyScan3D (Proto3000 Inc., Vaughan, ON, Canada). The plantar profiles from the foam box were then extracted by the scanner, as shown in Figure 1b. Next, the scanned data of both feet were preprocessed in reverse engineering software, Rapidform XOR (INUS Technology Ltd., Seoul, Korea). Then, the processed data were input to the computer-aided design (CAD) software, isoleCAD (Nmotion Orthotic Lab, Knoxville, TN, USA) to generate orthotic designs (Figure 1c).

### 2.3. Generating Orthotic Insole Design

**Figure 2:**
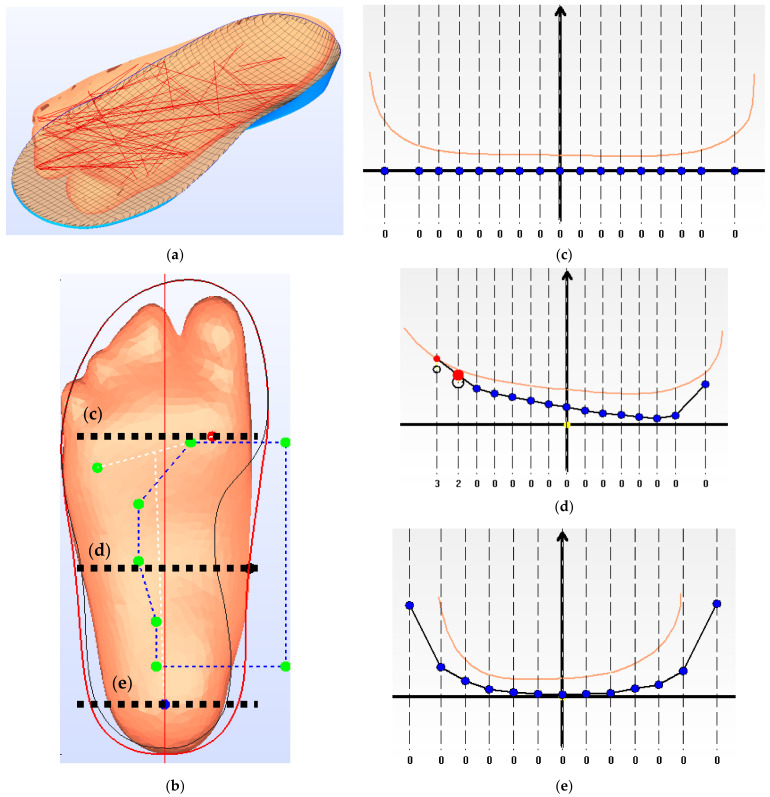
Design profile of the 3D printed insole: (**a**) An illustration of the fitting between the scanned 3D plantar foot profile and the insole; (**b**) Insole design profile based on the silhouette of the scanned plantar foot; cross-sectional design profiles at the (**c**) metatarsals; (**d**) apex of the medial longitudinal arch; and (**e**) heel center. The blue grid lines in (**b**) represent the arch support region identified by the software for design rectification; The orange lines in (**c**–**e**) represents the scanned plantar foot profile. The blacklines with blue dots in (**c**–**e**) represent the profile of the 3D printed insoles. The red and black circles in (**d**) represent the insole profile for the undercut (U+) and without undercut (U−) designs, respectively. The scale under (**c**–**e**) represent the difference in vertical displacement (in mm) between the automatic rectification and manual rectification, while the left side of the axis represents the medial direction.

The orthotic insole design encompassed both subject-specific customized features and rectified features. For the pattern layout of the insole, the boundary was extracted from the silhouette of the subject-specific 3D scanning. Based on the scanned plantar contour of the foot, a smooth interpolated 3D surface was generated as the design of the plantar contact surface of the insole, while the forefoot region of the surface was flattened. The heel and arch support regions were identified and rectified. The heel region was imposed with a heel cup (18 mm depth) for all the insole conditions, while R+U+ was given an additional elevation (3 mm) at the arch support area [22]. Figure 2 illustrates an example of the foot scan with rectified designs of the insole and the cross-sectional profiles.

The CAD software, isoleCAD, facilitated a semiautomatic design procedure, from the scanned 3D plantar contour data into a ready-to-build insole CAD file. In terms of the procedure, first of all, the users assisted the software in locating the first metatarsal head, fifth metatarsal head, and the heel center. Secondly, the users input the shoe size and the required insole thickness. We assigned an insole thickness of 3 mm, which is the usual thickness of insole materials [23]. Thirdly, based on the scanned 3D contour, the software generated the insole design with arch support, metatarsal pad, and heel cup. Finally, the design parameters could be adjusted in the CAD software, while the material settings were configurated into the 3D printer software.

**Figure 3:**
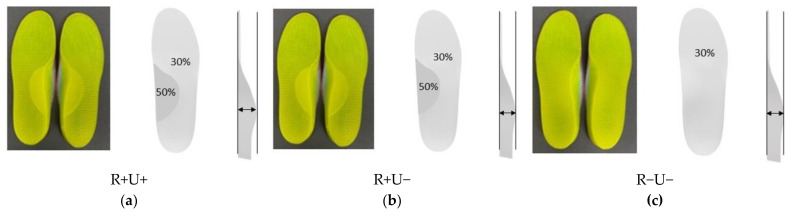
Schematics of the three orthotic insole designs. (**a**) R+U+, reinforced and undercut arch support insole; (**b**) R+U−, reinforced arch support insole without undercut; and (**c**) R−U−, insole without reinforced and undercut arch support.

The overall infill rate for the insole material (thermoplastic polyurethane, TPU) was 30%. In this study, we increased the infill rate of the arch support region to 50% for the R+ conditions. Apart from the control condition (no insole), we had three insole conditions (Figure 3) in this study: (1) reinforced and undercut arch support (R+U+); (2) reinforced without undercut arch support (R+U−); (3) without reinforced and undercut arch support (R−U−). The completed CADs were passed to the 3D printing software (Figure 1d).

### 2.4. Fabrication of 3D Printed Insole

According to the design and material infill settings of the CAD file, the orthotic insoles were printed using a fused deposition modeling (FDM) 3D printer (iSun3D Flx2, eSUN Industrial Co. Ltd., Shenzhen, China). The printer consisted of a nozzle of 0.8 mm diameter at an average operating temperature of 235 °C. We used TPU (eTPU-95A, Shenzhen Esun Industrial Co. Ltd., Shenzhen, China) for the insole material and printed at 0.4 mm layer thickness with a triangle pattern. There were three layers for the perimeters but no solid infill top and bottom layers for sake of breathability. The fabricated insoles are illustrated in Figure 3.

The arch support region was printed at a 30% or 50% infill rate, depending on the insole conditions. Therefore, we evaluated the difference in elasticity between the two infill rates using a mechanical testing machine (Instron AG-IS, Shimadzu, Japan). We printed five cylindrical specimens for each infill rate (30% and 50%) and loaded at 1mm/min compression speed. The height and diameter of the specimens were 25.4 mm and 12.7 mm, respectively, according to the ASTM D695 standard. The elastic moduli were 15.0 MPa (SD: 0.35) and 18.2 MPa (SD: 0.4), respectively for 30% and 50% infill rates.

### 2.5. Experimental Evaluation

The participants were invited for gait analysis with 3D printed insoles in the locomotion laboratory. The laboratory was equipped with an eight-camera motion capture system (Vicon, Oxford Metrics Ltd., Oxford, UK) and force platforms (OR6, AMTI, Watertown, MA, USA). The sampling frequencies were 100 Hz and 1000 Hz. The marker trajectory data collected by the motion capture system were filtered using the low-pass Butterworth at a cut-off frequency of 6 Hz, whereas that of the force platform was 300 Hz. In addition, the in-shoe plantar pressure measurement system (Pedar-X System, Novel Gmbh Inc., Munich, Germany) was available in the laboratory for the experiment. The motion capture system and the plantar pressure system were calibrated before the experiment.

**Figure 4:**
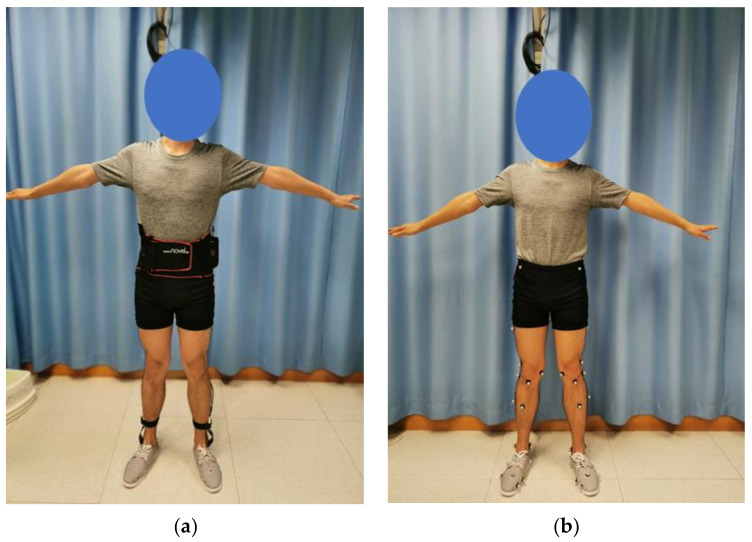
An illustration of the experimental setting of the participant: (**a**) wearing the in-shoe plantar pressure measurement system; (**b**) with infrared reflective markers attached for motion capture.

Twenty-nine infrared reflective markers were attached to the lower limbs of the participants according to the marker set of the Oxford Foot Model [24] for the motion capture, as shown in Figure 4. For all the insole conditions and the control, all participants wore the same type of canvas shoes at their own shoe size with holes for marker placement. We believed that canvas shoes possessed minimal features that could interfere with the results. The participants were given some time to familiarize themselves with the footwear and the insole conditions between sessions. For each insole condition, the participants were asked to walk at their comfortable speed along the straight walkway embedded with the force platform. A walking trial was regarded as successful if the footfalls were clean on the force platforms. We collected five walking trials for each condition. The first and last trials were discarded from analysis to minimize irregularities due to the initialization and termination of the experiment [25]. Only the right stride on the force platform was analyzed.

The same set of experiments was repeated using the in-shoe plantar pressure measurement system without the motion capture system and force platforms (Figure 4).

The order of the four conditions, (1) reinforced and undercut arch support (R+U+); (2) reinforced without undercut arch support (R+U−); (3) without reinforced and undercut arch support (R−U−); and (4) control without insole, were randomized (randomized crossover design). A 5 min break was given between conditions.

### 2.6. Outcome Measures and Statistical Analyses

The kinematic and kinetic data were processed in the software, Nexus 2.11 (Vicon, Oxford Metrics Ltd., Oxford, UK). The foot kinematics, including the peak hindfoot eversion, peak hindfoot (hindfoot-tibia) internal rotation, peak ankle (hindfoot-tibia) dorsiflexion, peak forefoot (forefoot-hindfoot) abduction, peak tibial internal rotation, were analyzed, in addition to the vertical ground reaction force.

The mask of the plantar pressure measurement was divided into medial and lateral regions transversely and into forefoot, midfoot, and hindfoot regions longitudinally, according to a protocol [26]. There were six regions after mask division. The peak pressure and pressure time integral for each region were analyzed.

Data for the successful trials were averaged before statistical analysis. Statistical analysis was performed using R Statistical Software (Foundation for Statistical Computing, Vienna, Austria). Since some data violated the normality assumption and some with outliers, we decided to unify all statistical analyses using the nonparametric test, Friedman test with the pairwise Wilcoxon signed-rank tests. No adjustment for multiple comparisons was made for this pilot study. The significance level was set at *p* = 0.05. The results were illustrated using box and whisker plots. The lower and upper hinges of the box represent the first and third quartiles, while the middle line represents the median (middle quartile). The upper whiskers are calculated based on the largest observation less than or equal to the upper hinge plus 1.5 times the interquartile range [27]. Data beyond the whiskers were regarded as outliers.

## 3. Results

### 3.1. Foot Kinematics

**Figure 5:**
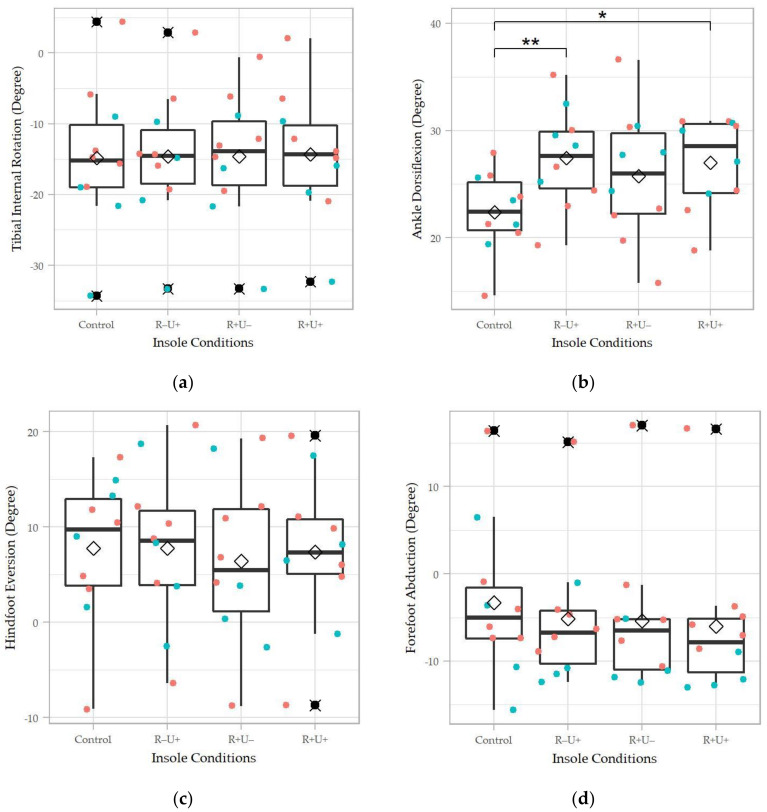
Foot kinematic parameters of the four insole conditions: (**a**) peak tibial internal rotation; (**b**) peak ankle dorsiflexion; (**c**) peak hindfoot eversion; and (**d**) peak forefoot abduction. R+U+: reinforced and undercut arch support insole; R+U−: reinforced arch support insole without undercut; R−U−: insole without reinforced and undercut arch support. Significance levels (* *p <* 0.05 and ** *p* < 0.01) refer to matched-pair comparison (Wilcoxon signed-rank test) between the specific insole conditions. ♢ represents the average of the data column; ⯍ represents outliers; teal colored dots represent data points of male participants; orange colored dots represent data points of female participants.

As shown in Figure 5, the peak ankle dorsiflexion demonstrated marginal significance (χ^2^(3) = 7.68, Kendell’s W = 0.256, *p* = 0.053). We found no evidence that the insole conditions were significantly different in the peak tibial internal rotation, hindfoot eversion, and forefoot abduction. However, there was a trend that the insole conditions reduced the hindfoot eversion and forefoot abduction.

The R−U+ and R+U+ conditions produced a significantly higher peak ankle dorsiflexion compared to the control (*p* = 0.01, *p* = 0.037). With respect to the tibial internal rotation, R−U+ was significantly higher than R+U+ (*p* = 0.036), despite that the Friedman test did not show a significant result in this parameter.

### 3.2. Vertical Ground Reaction Force

**Figure 6:**
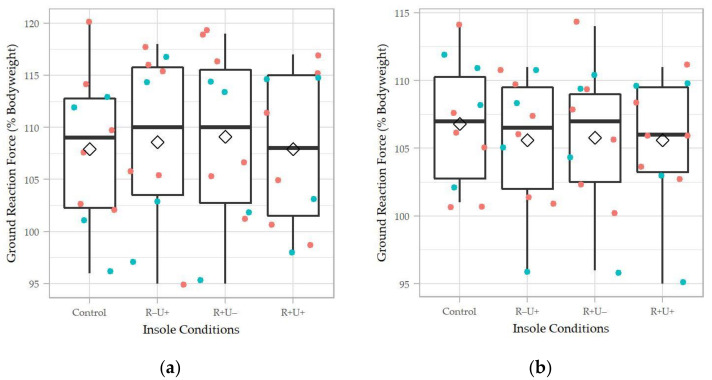
The (**a**) first peak and (**b**) second peak of ground reaction. R+U+: reinforced and undercut arch support insole; R+U−: reinforced arch support insole without undercut; R−U−: insole without reinforced and undercut arch support. Teal colored dots represent data points of male participants; orange colored dots represent data points of female participants.

As shown in Figure 6, there were no significant differences in the first and second peaks of the vertical ground reaction force (*p* > 0.05). From observation, R−U+ and R+U− seemed to produce a higher first peak of the vertical ground reaction force, while R+U+ seemed to be lower than the control. For the second peak vertical ground reaction force, all insole conditions appeared lower than the control.

### 3.3. Plantar Pressure

**Figure 7:**
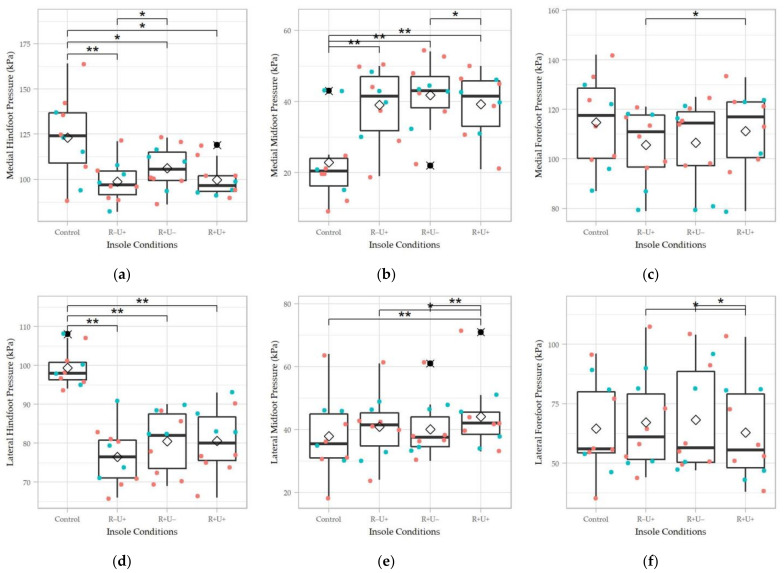
Peak pressure of the four insole conditions in different regions: (**a**) medial forefoot; (**b**) medial midfoot; (**c**) medial hindfoot; (**d**) lateral forefoot; (**e**) lateral midfoot; and (**f**) lateral hindfoot. R+U+: reinforced and undercut arch support insole; R+U−: reinforced arch support insole without undercut; R−U−: insole without reinforced and undercut arch support. Significance levels (* *p* < 0.05 and ** *p* < 0.01) refer to matched-pair comparison (Wilcoxon signed-rank test) between the specific insole conditions. ♢ represents the average of the data column; ⯍ represents outliers; Teal colored dots represent data points of male participants; orange colored dots represent data points of female participants.

Figure 7 shows the peak plantar pressure at different plantar regions. Except for the peak pressure of the medial forefoot region, the peak pressures of all other regions demonstrated significant differences between the insole conditions (*p* < 0.05). The effect size for the medial midfoot (χ^2^(3) = 21.5, Kendell’s W = 0.71, *p* < 0.001), lateral hindfoot (χ^2^(3) = 18.48, Kendell’s W = 0.61, *p* < 0.001), and lateral midfoot (χ^2^(3) = 16.5, Kendell’s W = 0.55, *p* < 0.001) regions were large, while that of the medial hindfoot (χ^2^(3) = 12.58, Kendell’s W = 0.42, *p* = 0.006) and lateral forefoot (χ^2^(3) = 9.97, Kendell’s W = 0.33, *p* = 0.02) were moderate.

For the pairwise comparison, the insole conditions (R+U+, R−U+, and R+U−) generally elevated the peak pressure at the medial midfoot region and reduced the peak pressure at the hindfoot compared to control (*p* < 0.05). Moreover, the R+U− was significantly higher than R+U+ and R−U+ for the peak medial midfoot pressure (*p* = 0.03) and medial hindfoot pressure (*p* = 0.01). For the lateral side, R+U+ had a significantly lower peak pressure at the forefoot (*p* = 0.01, *p* = 0.02), but higher at the midfoot, respectively, compared to that of R−U+ and R+U− (*p* = 0.02, *p* = 0.009).

**Figure 8:**
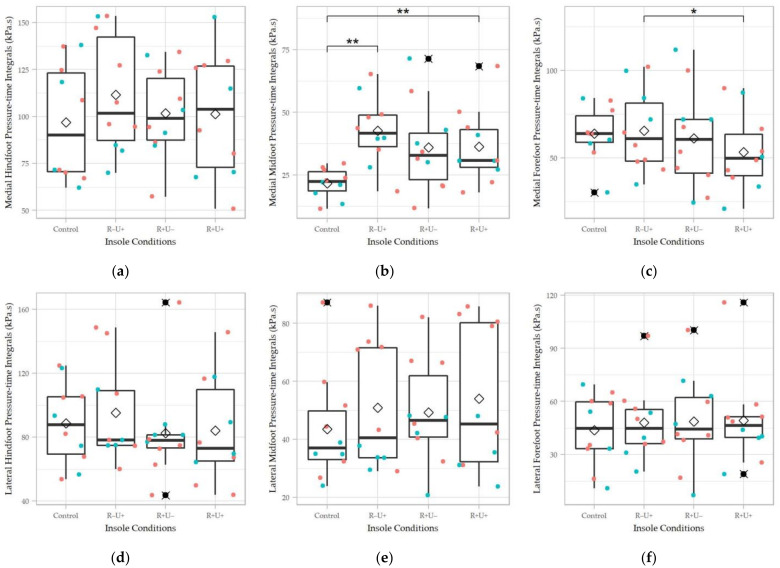
Pressure-time integrals of the four insole conditions in different regions: (**a**) medial forefoot; (**b**) medial midfoot; (**c**) medial hindfoot; (**d**) lateral forefoot; (**e**) lateral midfoot; and (**f**) lateral hindfoot. R+U+: reinforced and undercut arch support insole; R+U−: reinforced arch support insole without undercut; R−U−: insole without reinforced and undercut arch support. Significance levels (* *p* < 0.05 and ** *p* < 0.01) refer to matched-pair comparison (Wilcoxon signed-rank test) between the specific insole conditions. ♢ represents the average of the data column; ⯍ represents outliers; Teal colored dots represent data points of male participants; orange colored dots represent data points of female participants.

For the pressure-time integral, significant difference was only found at the medial midfoot region (χ^2^(3) = 8.76, Kendell’s W = 0.29, *p* = 0.03) in the Friedman test, while R−U+ (*p* = 0.004) and R+U+ (*p* = 0.004) were significantly different from the control in the pairwise comparison (Figure 8). Besides, R+U+ had a significantly lower pressure-time integral at the medial forefoot region than R−U+ (*p* = 0.03) despite that the omnibus test did not demonstrate significant difference. For the other regions, the insole conditions seemed to elevate the pressure-time integral at the medial hindfoot and lateral midfoot regions by observation.

## 4. Discussion

The technological advancements in morphology acquisition and 3D printing enable the digitalization for customized orthotics with improved accuracy and thus better rehabilitation [16]. Therefore, the technique could probably replace the traditional manufacturing of orthoses in the future [17]. The 3D printing technology had been taken advantage of by the total contact insoles for diabetes, matching the foot morphology to the insole for pressure relief [28]. However, the technique may not be sufficient for tackling foot deformities. A rectification process was essential for biomechanical control or correction. Some studies have attempted to use a plantar pressure-based method to drive the rectification of the design parameters [29]. This study aimed to evaluate two crucial design parameters (i.e., the reinforced stiffness at the arch support, and undercut of the arch support height) of foot orthosis for flatfoot to provide evidence for assisting the rectification process.

Controlling foot pronation with medial longitudinal arch support is the goal of orthotic functions for flatfooted individuals, strongly associated with hindfoot eversion, forefoot abduction, and tibial internal rotation [30]. Flatfooted individuals with pronated feet generally possessed an everted calcaneus, abducted forefoot, and a greater tibial internal rotation [30]. Suppressing the calcaneal eversion was believed to be the most important function achieved by orthotic insoles [31]. The 3D printed insoles fabricated by Mo, et al. [32] and Telfer, et al. [33] also demonstrated a significant reduction in peak hindfoot eversion. Our study found no significant change but a slight trend of reduction on the hindfoot eversion and forefoot abduction. One plausible reason was that the heel cup was insufficient to secure the hindfoot and counteract pronation tendency [34,35]. Wahmkow, et al. [36] discovered that arch support insoles had no effect on hindfoot eversion and internal tibial rotation, probably due to the high subject variation. On the other hand, although a meta-analysis substantiated the function of foot orthoses on hindfoot eversion control, insoles with arch support design showed no significant effect in the subgroup analysis [12]. Insole postings were suggested to be effective [12]. However, the levels and configuration were difficult to determine clinically or based on the subject morphology from the 3D scan that warranted further investigations.

Ankle dorsiflexion was elevated in our insole conditions, though existing literature presented mixed results and implications. One faction proposed that flatfootedness was sourced by excessive dorsiflexion and eversion leading to talotarsal dislocation and pronation [37], in which ankle dorsiflexion or hypermobility should be controlled. Another faction supported the proposition that the pronated foot with everted hindfoot was compensated by the limited range of motion at the ankle joint, which should be recovered [38]. Therefore, conflicting results on ankle dorsiflexion towards the use of insoles were found in the literature [39,40]. We may learn that the underlying mechanism and flexibility of the flatfoot will be recognized and considered in the orthotic design in addition to the foot morphology and severity of the deformity.

Ground reaction force was seldom used for orthosis evaluation, and we found no significant difference between the insole conditions in our study. Ng, et al. [41] investigated the use of prefabricated foot orthosis in flatfooted athletes and found a significant increase in impact force and loading rate with the foot orthosis. The authors attributed the effects to the geometry and cushioning of the insole. It was reasonable that the loading rate was attenuated by the cushioning; nonetheless, we believed that the explanation was not very convincing for the findings in impact force.

The rectification process plays an important role in altering the plantar pressure to control the biomechanical environment of the plantar foot. Offloading high pressure was advocated by the 3D printed total contact insole [42], which was partially supported by our findings. The orthotic insoles in our study significantly reduced the peak pressure of the hindfoot, although the effects on pressure-time integrals were not significant. An uncut arch support insole without reinforcement (R−U+) appeared to be more promising in unloading the pressure at the hindfoot regions. The 3D printed heel support insoles fabricated by Jin, et al. [43] for healthy individuals and the 3D printed insoles fabricated by Tarrade, et al. [44] for workers also found a significant reduction in peak pressure over the hindfoot region. It was anticipated that the midfoot pressures were increased because of the arch support, which is consistent with other similar studies [42,43,44,45].

Reinforced arch support with an undercut (R+U+) should have, intuitively, given an immense amount of support. However, R+U+ did not produce the highest peak pressure at the medial midfoot but substantially high peak pressure at the lateral midfoot. This result could be due to over-impinging on the medial longitudinal arch. Besides, an undercutting arch support without reinforcement (R−U+) appeared to have higher support over time, as revealed by the pressure-time integral over the medial midfoot region. Therefore, along with offloading of the hindfoot, we recommended that R−U+ could be a better option among the three 3D-printed insoles.

There were several methods to acquire the plantar foot shape for the fabrication of customized foot orthoses. Traditionally, orthotists take a plaster cast from the participants, commonly at prone lying position, while the use of the impression foam box provides a more convenient way to obtain the plantar foot shape at partial weight-bearing [21]. The 3D scanners can now achieve the digitalization of a cast. In this study, we took a 3D scan from the impressed foam box since this could facilitate a more stable casting in addition to the control of subtalar joint neutral and partial weight-bearing. Nevertheless, the decision was also primarily subject to the constraint of our handheld scanning device that required a flat surface during the scan for calibration. A few studies found that the scanned foam box approach produced excellent reliability for foot lengths and widths [21,46,47]. However, a direct 3D surface scan on the plantar foot could further improve the reliability of the scan data, particularly at the medial arch region and forefoot-to-rearfoot alignment [21].

Evaluating the fitness or effectiveness of the customized insoles remains one of the challenges. In clinical practice, orthotists match the profile of the insole with the plantar foot by observation and make the necessary adjustment in the fitting process. The traditional fabrication and fitting process is subjective and highly dependent on empirical experience and craftmanship [16,48], while the development of CAD/CAM, particularly the 3D printing techniques, can improve the reliability and objectivity of the orthotics services [21]. Therefore, we do assume that the geometrical fitness of the customized insoles was adequately achieved. However, it shall be noted that the geometrical fitness or matching of the plantar foot profile may not necessarily always be the primary aim or index for orthotics from a clinical point of view. On the one hand, plantar shape customization with good geometrical fitness is essential for pressure offloading purposes in some foot pathologies, such as neuropathic foot ulceration and pes cavus (high arch) [21,48]. On the other hand, some foot pathologies, such as flatfoot, may have a collapsed or malpositioned foot structure in which customizing the original plantar foot shape may not be adequate to achieve the “therapeutic” effects. Hence, the rectification process is crucial. Controlling the patients’ physical conditions, such as the joint alignment and weight-bearing state during casting, plays a vital role, aside from the geometrical fitness and maintaining the subtalar joint neutral being a rule of thumb [49]. To evaluate whether the orthosis could adjust the muscular action and internal load transfer, some studies measured the surface electromyography [50] while computer simulations were also utilized to predict the muscle force, internal joint loading, and the stress of the plantar fascia and ligaments [51,52,53].

There were some limitations in this research. First of all, the sample size of this study was small (*n* = 10), such that the findings lacked generalizability and were confined to a preliminary exploratory study. We anticipated that increasing the sample size might qualify sufficient statistical power to achieve our hypothesis. We also did not apply *p*-value adjustment for multivariate and pairwise comparisons in this exploratory study, which may yield some familywise errors. In addition, the interaction effect was not investigated. Secondly, we recruited only university students with mild flexible flatfoot in this study. In fact, children, elderly, and overweight individuals have a high frequency of flatfootedness [3,54]. These populations could have different foot morphologies, underlying mechanisms, and severities of flatfootedness [3,54], apart from the flexible and rigid flatfoot type. For example, posterior tibial tendon dysfunction was proven the primary cause of the flatfoot deformity in both clinical and biomechanical studies [55,56]. In contrast, the adult-acquired flatfoot of the elderly could be compounded with morphological changes in physiological aging [54] and a higher prevalence of other foot deformities [57], in which 3D printed insoles or footwear may better address the poor fitting problem. The feet of elderly people tend to be flatter and broader [58,59] and have a medialized plantar pressure [60,61]. Han, et al. [62] fabricated arch support insoles for the elderly with flatfootedness and discovered that the insoles elevated the peak pressure of the medial midfoot region, which aligned with the trend of our study. More studies on elderly flatfooted individuals are warranted. There may be other causes or factors, such as symptomatic/asymptomatic, injury, arthritis, neuropathy, and footwear, generated different gait deviations [63,64] and anticipated different orthosis functions. Therefore, our findings and designs may not be conclusive to different types and populations.

In terms of the orthotic design, some design features and material selections were based on empirical experiences; despite that, some were cited from existing literature and evaluated. We only considered limited features in the study due to the cost of fabrication. More levels and design factors should also be incorporated for an established sensitivity analysis, particularly the incorporation of posting [12]. Future studies may utilize the Taguchi method to evaluate more design parameters with fewer factor combinations and thus cost, which have also been widely adopted in orthotic designs [65,66]. In addition, subject ratings, electromyography, musculoskeletal model, and finite element model can also be utilized to frame a comprehensive biomechanical profile (including muscle force and internal stress/strain) for the evaluation of 3D printed insole designs for flatfooted individuals [17,67,68].

## 5. Conclusions

The 3D printed insoles increased the peak pressure and pressure-time integral of the medial midfoot indicating support on the medial longitudinal arch, accompanied with an offloading on the hindfoot and greater ankle dorsiflexion. In addition, the insoles tended to control hindfoot eversion and forefoot abduction, although insignificant. The reinforced undercutting arch support insole (R+U+) did not necessary producing more positive results probably due to over-cutting impingement. Studies with a larger sample size are warranted with stratified flatfooted features/types and adjusted for the ankle flexibility and evaluate with more insole design features, particularly insole postings.

## Data Availability

Data are contained within the article.

## References

[1] Pinney S.J., Lin S.S. (2006). Current concept review: Acquired adult flatfoot deformity. Foot Ankle Int..

[2] Aenumulapalli A., Kulkarni M.M., Gandotra A.R. (2017). Prevalence of flexible flat foot in adults: A cross-sectional study. J. Clin. Diagn. Res. JCDR.

[3] Sadeghi-Demneh E., Jafarian F., Melvin J.M., Azadinia F., Shamsi F., Jafarpishe M. (2015). Flatfoot in school-age children: Prevalence and associated factors. Foot Ankle Spec..

[4] Harris E.J., Vanore J.V., Thomas J.L., Kravitz S.R., Mendelson S.A., Mendicino R.W., Silvani S.H., Gassen S.C. (2004). Diagnosis and treatment of pediatric flatfoot. J. Foot Ankle Surg..

[5] Benedetti M.G., Ceccarelli F., Berti L., Luciani D., Catani F., Boschi M., Giannini S. (2011). Diagnosis of flexible flatfoot in children: A systematic clinical approach. Orthopedics.

[6] Cacace L.A., Hillstrom H.J., Dufour A.B., Hannan M.T. (2013). The association between pes planus foot type and the prevalence of foot disorders: The Framingham foot study. Osteoarthr. Cartil..

[7] Gross K.D., Felson D.T., Niu J., Hunter D.J., Guermazi A., Roemer F.W., Dufour A.B., Gensure R.H., Hannan M.T. (2011). Association of flat feet with knee pain and cartilage damage in older adults. Arthritis Care Res..

[8] Tang C.Y.K., Ng K.H., Lai J. (2020). Adult flatfoot. BMJ.

[9] Neville C.G., Houck J.R. (2009). Choosing among 3 ankle-foot orthoses for a patient with stage II posterior tibial tendon dysfunction. J. Orthop. Sports Phys. Ther..

[10] Flemister A.S., Neville C.G., Houck J. (2007). The Relationship Between Ankle, Hindfoot, and Forefoot Position and Posterior Tibial Muscle Excursion. Foot Ankle Int..

[11] Chou L.B., Wapner K.L., Coughlin M.J., Saltzman C.L. (2014). Conservative Treatment of the Foot. Mann’s Surgery of the Foot and Ankle.

[12] Desmyttere G., Hajizadeh M., Bleau J., Begon M. (2018). Effect of foot orthosis design on lower limb joint kinematics and kinetics during walking in flexible pes planovalgus: A systematic review and meta-analysis. Clin. Biomech..

[13] Dars S., Uden H., Banwell H.A., Kumar S. (2018). The effectiveness of non-surgical intervention (Foot Orthoses) for paediatric flexible pes planus: A systematic review: Update. PLoS ONE.

[14] Tang S.F.-T., Chen C.-H., Wu C.-K., Hong W.-H., Chen K.-J., Chen C.-K. (2015). The effects of total contact insole with forefoot medial posting on rearfoot movement and foot pressure distributions in patients with flexible flatfoot. Clin. Neurol. Neurosurg..

[15] Su S., Mo Z., Guo J., Fan Y. (2017). The effect of arch height and material hardness of personalized insole on correction and tissues of flatfoot. J. Healthc. Eng..

[16] Barrios-Muriel J., Romero-Sánchez F., Alonso-Sánchez F.J., Rodriguez Salgado D. (2020). Advances in orthotic and prosthetic manufacturing: A technology review. Materials.

[17] Choo Y.J., Boudier-Revéret M., Chang M.C. (2020). 3D printing technology applied to orthosis manufacturing: Narrative review. Ann. Palliat. Med..

[18] Davia-Aracil M., Hinojo-Pérez J.J., Jimeno-Morenilla A., Mora-Mora H. (2018). 3D printing of functional anatomical insoles. Comput. Ind..

[19] Staheli L.T., Chew D.E., Corbett M. (1987). The longitudinal arch. A survey of eight hundred and eighty-two feet in normal children and adults. J. Bone Joint Surg. Am. Vol..

[20] Riccio I., Gimigliano F., Gimigliano R., Porpora G., Iolascon G. (2009). Rehabilitative treatment in flexible flatfoot: A perspective cohort study. Musculoskelet. Surg..

[21] Telfer S., Gibson K.S., Hennessy K., Steultjens M.P., Woodburn J. (2012). Computer-aided design of customized foot orthoses: Reproducibility and effect of method used to obtain foot shape. Arch. Phys. Med. Rehabil..

[22] Scherer P.R., Werd M.B., Knight E.L. (2010). Custom Foot Orthoses. Athletic Footwear and Orthoses in Sports Medicine.

[23] Mendes A.A.M.T., de Almeida Silva H.J., Costa A.R.A., Pinheiro Y.T., de Almeida Lins C.A., de Souza M.C. (2020). Main types of insoles described in the literature and their applicability for musculoskeletal disorders of the lower limbs: A systematic review of clinical studies. J. Bodyw. Mov. Ther..

[24] Carson M., Harrington M., Thompson N., O’connor J., Theologis T. (2001). Kinematic analysis of a multi-segment foot model for research and clinical applications: A repeatability analysis. J. Biomech..

[25] Wong D.W.-C., Lam W.K., Yeung L., Lee W.C. (2015). Does long-distance walking improve or deteriorate walking stability of transtibial amputees?. Clin. Biomech..

[26] Ma C.Z.-H., Zheng Y.-P., Lee W.C.-C. (2018). Changes in gait and plantar foot loading upon using vibrotactile wearable biofeedback system in patients with stroke. Top. Stroke Rehabil..

[27] McGill R., Tukey J.W., Larsen W.A. (1978). Variations of box plots. Am. Stat..

[28] Ahmed S., Barwick A., Butterworth P., Nancarrow S. (2020). Footwear and insole design features that reduce neuropathic plantar forefoot ulcer risk in people with diabetes: A systematic literature review. J. Foot Ankle Res..

[29] Jiang Y., Wang D., Ying J., Chu P., Qian Y., Chen W. (2021). Design and Preliminary Validation of Individual Customized Insole for Adults with Flexible Flatfeet Based on the Plantar Pressure Redistribution. Sensors.

[30] Levinger P., Murley G.S., Barton C.J., Cotchett M.P., McSweeney S.R., Menz H.B. (2010). A comparison of foot kinematics in people with normal- and flat-arched feet using the Oxford Foot Model. Gait Posture.

[31] Kido M., Ikoma K., Hara Y., Imai K., Maki M., Ikeda T., Fujiwara H., Tokunaga D., Inoue N., Kubo T. (2014). Effect of therapeutic insoles on the medial longitudinal arch in patients with flatfoot deformity: A three-dimensional loading computed tomography study. Clin. Biomech..

[32] Mo S., Leung S.H., Chan Z.Y., Sze L.K., Mok K.-M., Yung P.S., Ferber R., Cheung R.T. (2019). The biomechanical difference between running with traditional and 3D printed orthoses. J. Sports Sci..

[33] Telfer S., Abbott M., Steultjens M.P., Woodburn J. (2013). Dose–response effects of customised foot orthoses on lower limb kinematics and kinetics in pronated foot type. J. Biomech..

[34] McCulloch M.U., Brunt D., Vander Linden D. (1993). The effect of foot orthotics and gait velocity on lower limb kinematics and temporal events of stance. J. Orthop. Sports Phys. Ther..

[35] Aminian G., Safaeepour Z., Farhoodi M., Pezeshk A.F., Saeedi H., Majddoleslam B. (2013). The effect of prefabricated and proprioceptive foot orthoses on plantar pressure distribution in patients with flexible flatfoot during walking. Prosthet. Orthot. Int..

[36] Wahmkow G., Cassel M., Mayer F., Baur H. (2017). Effects of different medial arch support heights on rearfoot kinematics. PLoS ONE.

[37] Needleman R.L. (2005). Current topic review: Subtalar arthroereisis for the correction of flexible flatfoot. Foot Ankle Int..

[38] Hösl M., Böhm H., Multerer C., Döderlein L. (2014). Does excessive flatfoot deformity affect function? A comparison between symptomatic and asymptomatic flatfeet using the Oxford Foot Model. Gait Posture.

[39] Salles A.S., Gyi D.E. (2013). An evaluation of personalised insoles developed using additive manufacturing. J. Sports Sci..

[40] Chen Y.-C., Lou S.-Z., Huang C.-Y., Su F.-C. (2010). Effects of foot orthoses on gait patterns of flat feet patients. Clin. Biomech..

[41] Ng J.W., Chong L.J., Pan J.W., Lam W.-K., Ho M., Kong P.W. (2021). Effects of foot orthosis on ground reaction forces and perception during short sprints in flat-footed athletes. Res. Sports Med..

[42] Telfer S., Woodburn J., Collier A., Cavanagh P. (2017). Virtually optimized insoles for offloading the diabetic foot: A randomized crossover study. J. Biomech..

[43] Jin H., Xu R., Wang S., Wang J. (2019). Use of 3D-Printed Heel Support Insoles Based on Arch Lift Improves Foot Pressure Distribution in Healthy People. Med. Sci. Monit..

[44] Tarrade T., Doucet F., Saint-Lô N., Llari M., Behr M. (2019). Are custom-made foot orthoses of any interest on the treatment of foot pain for prolonged standing workers?. Appl. Ergon..

[45] Huang Y.-P., Peng H.-T., Wang X., Chen Z.-R., Song C.-Y. (2020). The arch support insoles show benefits to people with flatfoot on stance time, cadence, plantar pressure and contact area. PLoS ONE.

[46] Laughton C., McClay Davis I., Williams D.S. (2002). A comparison of four methods of obtaining a negative impression of the foot. J. Am. Podiatr. Med. Assoc..

[47] Carroll M., Annabell M.-E., Rome K. (2011). Reliability of capturing foot parameters using digital scanning and the neutral suspension casting technique. J. Foot Ankle Res..

[48] Jandova S., Mendricky R. (2021). Benefits of 3D Printed and Customized Anatomical Footwear Insoles for Plantar Pressure Distribution. 3d Print. Addit. Manuf..

[49] Ball K.A., Afheldt M.J. (2002). Evolution of foot orthotics—Part 1: Coherent theory or coherent practice?. J. Manip. Physiol. Ther..

[50] Barn R., Brandon M., Rafferty D., Sturrock R.D., Steultjens M., Turner D.E., Woodburn J. (2014). Kinematic, kinetic and electromyographic response to customized foot orthoses in patients with tibialis posterior tenosynovitis, pes plano valgus and rheumatoid arthritis. Rheumatology.

[51] Peng Y., Wang Y., Wong D.W.-C., Chen T.L.-W., Zhang G., Tan Q., Zhang M. (2021). Extrinsic foot muscle forces and joint contact forces in flexible flatfoot adult with foot orthosis: A parametric study of tibialis posterior muscle weakness. Gait Posture.

[52] Peng Y., Wong D.W.-C., Wang Y., Chen T.L.-W., Tan Q., Chen Z., Jin Z., Zhang M. (2020). Immediate effects of medially posted insoles on lower limb joint contact forces in adult acquired flatfoot: A pilot study. Int. J. Environ. Res. Public Health.

[53] Peng Y., Wong D.W.-C., Wang Y., Chen T.L.-W., Zhang G., Yan F., Zhang M. (2021). Computational models of flatfoot with three-dimensional fascia and bulk soft tissue interaction for orthosis design. Med. Nov. Technol. Devices.

[54] Nozaki S., Watanabe K., Teramoto A., Kamiya T., Katayose M., Ogihara N. (2021). Sex-and age-related variations in the three-dimensional orientations and curvatures of the articular surfaces of the human talus. Anat. Sci. Int..

[55] Ross M.H., Smith M.D., Vicenzino B. (2017). Reported selection criteria for adult acquired flatfoot deformity and posterior tibial tendon dysfunction: Are they one and the same? A systematic review. PLoS ONE.

[56] Wong D.W.-C., Wang Y., Leung A.K.-L., Yang M., Zhang M. (2018). Finite element simulation on posterior tibial tendinopathy: Load transfer alteration and implications to the onset of pes planus. Clin. Biomech..

[57] Mickle K.J., Munro B.J., Lord S.R., Menz H.B., Steele J.R. (2010). Foot shape of older people: Implications for shoe design. Footwear Sci..

[58] Luo X.D., Xue C.-H., Li Y. (2017). Study on the foot shape characteristics of the elderly in China. Foot.

[59] Tomassoni D., Traini E., Amenta F. (2014). Gender and age related differences in foot morphology. Maturitas.

[60] Chiu M.-C., Wu H.-C., Chang L.-Y., Wu M.-H. (2013). Center of pressure progression characteristics under the plantar region for elderly adults. Gait Posture.

[61] Kernozek T., LaMott E. (1995). Comparisons of plantar pressures between the elderly and young adults. Gait Posture.

[62] Han K.-H., Bae K.-H., Jung H.-G., Ha M.-S., Choi D.-Y., Lee J.-S., Yang J.-O. (2018). Comparison of plantar pressure and COP parameters in three types of arch support insole during stair descent in elderly with flatfoot. J. Korean Appl. Sci. Technol..

[63] Kerr C., Zavatsky A., Theologis T., Stebbins J. (2019). Kinematic differences between neutral and flat feet with and without symptoms as measured by the Oxford foot model. Gait Posture.

[64] Shin H.S., Lee J.H., Kim E.J., Kyung M.G., Yoo H.J., Lee D.Y. (2019). Flatfoot deformity affected the kinematics of the foot and ankle in proportion to the severity of deformity. Gait Posture.

[65] Cheung J.T.-M., Zhang M. (2008). Parametric design of pressure-relieving foot orthosis using statistics-based finite element method. Med Eng. Phys..

[66] Zhang H., Lv M.L., Yang J., Niu W., Cheung J.C.-W., Sun W., Wong D.W.-C., Ni M. (2020). Computational modelling of foot orthosis for midfoot arthritis: A Taguchi approach for design optimization. Acta Bioeng. Biomech..

[67] Peng Y., Wong D.W.-C., Chen T.L.-W., Wang Y., Zhang G., Yan F., Zhang M. (2021). Influence of arch support heights on the internal foot mechanics of flatfoot during walking: A muscle-driven finite element analysis. Comput. Biol. Med..

[68] Yoon J.-G., Yoo K.-T., Lee J.-H., Park J.-M., Min K.-O., Choi J.-H. (2012). The analysis of Lower Limb Muscle Activity and Motion Analysis according to Normal Foot and Flatfoot during Walking. J. Int. Acad. Phys. Ther. Res..

